# Parenting Stress and Adherence to Occlusion Therapy in the Infant Aphakia Treatment Study: A Secondary Analysis of a Randomized Clinical Trial

**DOI:** 10.1167/tvst.8.1.3

**Published:** 2019-01-02

**Authors:** Carolyn Drews-Botsch, Marianne Celano, George Cotsonis, Lindreth DuBois, Scott R. Lambert, for the Infant Aphakia Treatment Study Group

**Affiliations:** 1 Department of Epidemiology, Rollins School of Public Health, Emory University, Atlanta, GA, USA; 2 Department of Psychiatry & Behavioral Sciences, Emory University School of Medicine, Atlanta, GA, USA; 3 Department of Biostatistics and Bioinformatics, Rollins School of Public Health, Emory University, Atlanta, GA, USA; 4 Department of Ophthalmology, Emory University School of Medicine, Atlanta, GA, USA; 5 Department of Ophthalmology, Stanford University School of Medicine, Palo Alto, CA, USA

**Keywords:** unilateral congenital cataract, occlusion, adherence, parenting stress

## Abstract

**Purpose:**

Using data from the Infant Aphakia Treatment Study, we examined the relationship between adherence to patching and parenting stress.

**Methods:**

Caregivers completed the Parenting Stress Index 3 months after surgery (*n* = 106), after a visual acuity assessment at 12 months of age (*n* = 97), and at 4.25 (*n* = 96) years of age. Patching was reported in quarterly telephone interviews and annual 7-day patching diaries, and averaged across all assessments prior to and in the 6 months following the first stress assessment, and for 6 months before and after the other two stress assessments. The association was assessed using linear regression.

**Results:**

Caregivers reporting the highest stress levels 3 months after surgery (i.e., 75th percentile) subsequently reported approximately three-quarters (0.87, 95% confidence interval −1.3 to −0.34) of an hour a day less patching than caregivers reporting the least stress (i.e., the 25th percentile) after controlling for prior patching and other confounders. The association was in the same direction, but not statistically significant, after the second stress assessment and was not apparent at 4.25 years of age. In contrast to our hypothesis, we did not find evidence that higher levels of patching were associated with subsequent increases in parenting stress.

**Conclusions:**

Three months after surgery, higher levels of parenting stress are associated with poorer adherence to patching, and thus stress may contribute to early adherence to patching.

**Translational Relevance:**

Clinicians may wish to provide support to caregivers exhibiting high levels of stress since it may impact their ability to adhere to prescribed patching.

## Introduction

Infants born with visually significant unilateral cataracts often have poor visual outcomes. Previous reports suggest that achieving a good outcome requires early surgical removal of the cataract, consistent optical correction, and good adherence to a regimen of occlusion of the fellow eye.^1^–^5^ The caregiver provides a central role in achieving adherence to prescribed occlusion, particularly among infants.^6^ Although adherence to occlusion therapy is likely necessary for achieving good visual acuity, adherence to prescribed occlusion therapy is often difficult for caregivers, and previous reports in children prescribed occlusion therapy for amblyopia have suggested that patching is stressful for families^7^ and occlusion therapy may strain familial relationships and negatively impact the child-caregiver relationship.^8^ Further, such stress may contribute to poor adherence.^9^ However, the evidence of such negative effects is not universal, and some studies have shown that amblyopia treatment is not associated with negative psychosocial outcomes for patients or their caregivers.^10^

Additionally, much of the available evidence on the relationship between adherence to patching and parenting stress has focused on treatment of amblyopia among preschool- and early elementary school-aged children.^6^–^10^ Few of these studies have focused on children prescribed occlusion therapy for unilateral congenital cataract (UCC). Occlusion therapy prescribed for UCC is likely to be particularly vulnerable to parenting stress because the outcomes of such treatment are often poor,^11^ patching of up to 50% of a child's waking hours is often prescribed,^12^ treatment is initiated following surgery in early infancy, which may in itself be stressful for caregivers, and the prescribed patching continues for years. Thus, we felt it important to understand the relationship between parenting stress and adherence to patching in this population of young children.

**Figure 1:**
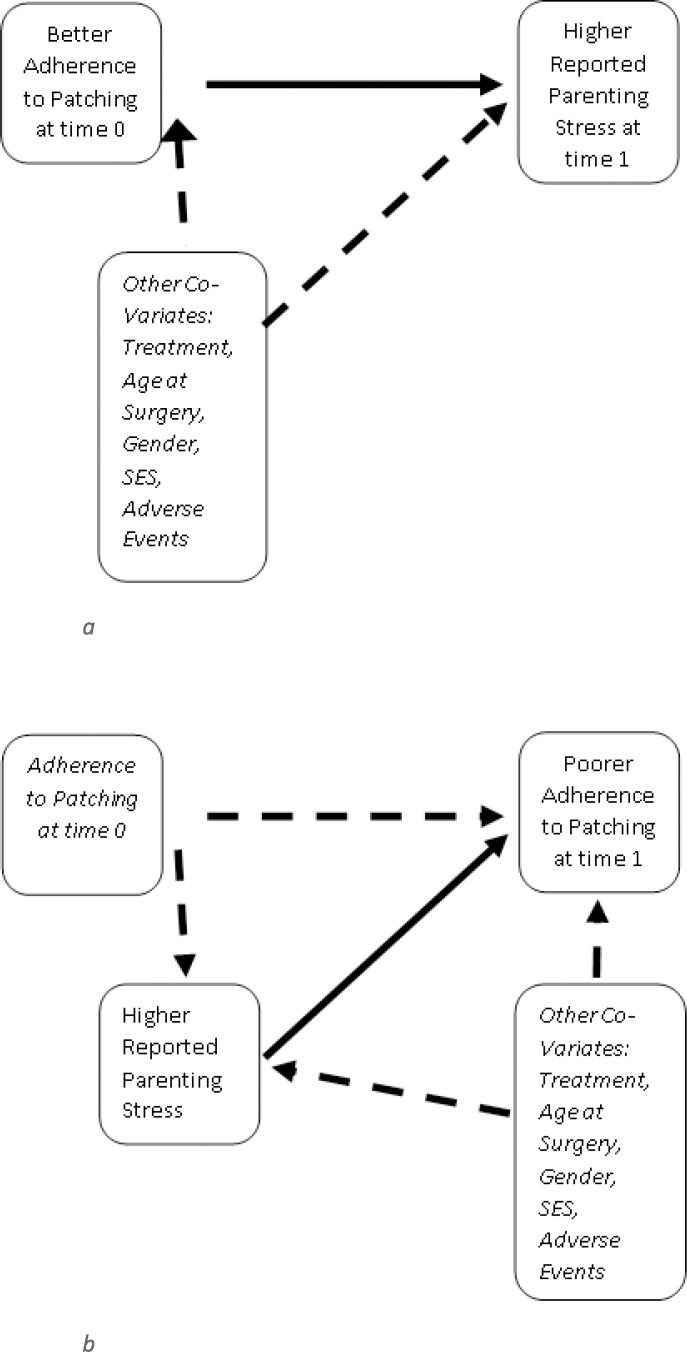
Hypothesized relationships between parenting stress and adherence to prescribed patching. (a) Hypothesis 1: Higher levels of adherence to patching increase stress associated with the parenting role. (b) Hypothesis 2: Higher levels of parenting stress reduce adherence to prescribed patching.

**Figure 2:**
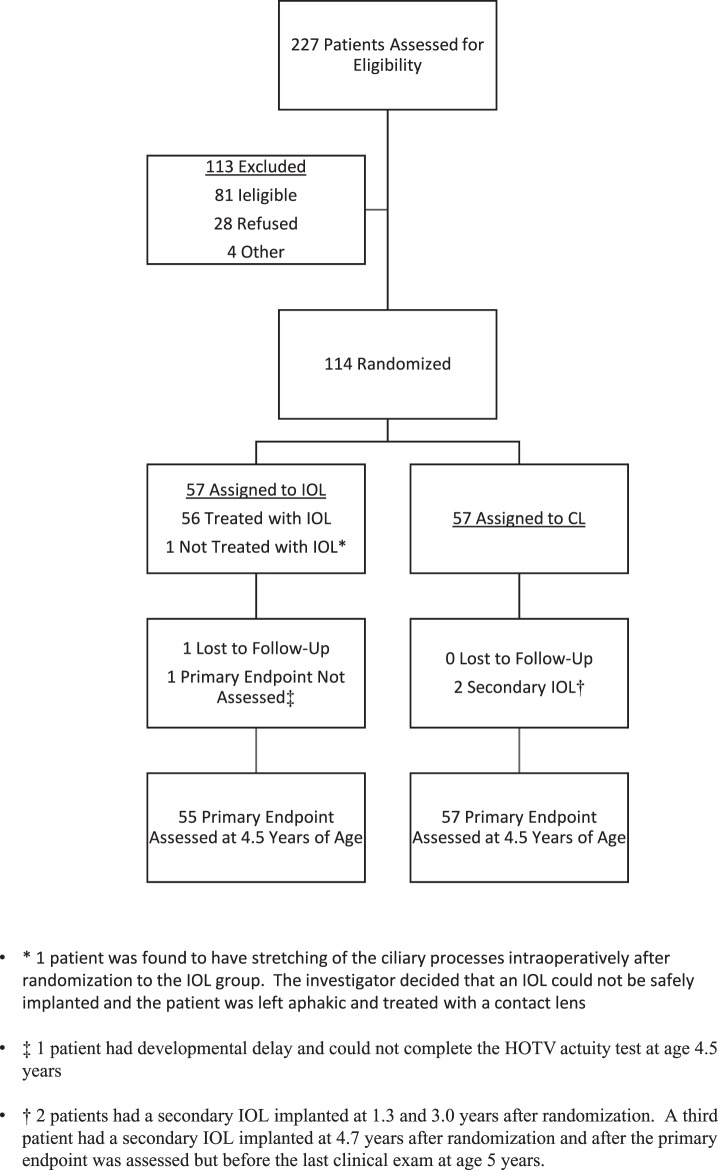
Consolidated Standards or Reporting Trials (CONSORT) diagram for the IATS.

The Infant Aphakia Treatment Study (IATS) is a multicenter, randomized, controlled clinical trial of treatment for UCC. The primary objective is to compare visual acuity in children with a UCC if an intraocular lens (IOL) is implanted at the time of cataract extraction with visual acuity in children left aphakic.^11^,^12^ The IATS has documented that such eyes achieve a wide variety of visual outcomes, but that visual acuity at 4.5 years of age does not differ by treatment group.^11^ Further, we have noted that among parents of children with unilateral cataracts, although parenting stress in the first months after surgery is not substantially higher than expected based on population norms, such stress is higher in parents whose children received an IOL than in those left aphakic,^13^,^14^ possibly because IOL implantation is associated with an increased risk of adverse events and reoperations in the first few postoperative months.^11^ However, the specific relationship between patching in children treated for UCC and parenting stress is not clear. It is possible that patching a child with a unilateral cataract is stressful for caregivers because they recognize the importance of occlusion therapy for the child's long-term visual outcomes while at the same time the child actively resists patching, particularly if the child has poor visual acuity in the treated eye. Thus, it is possible that parents who are able to patch their child for more time might report higher levels of parenting stress. Alternatively, or additionally, parents who have higher levels of either parenting stress or life stress may be less able to adhere to prescribed levels of patching. Thus, higher levels of reported stress would be associated with reduced adherence to prescribed patching. The current analysis examines both questions by hypothesizing that: (1) after controlling for other predictors of caregiver stress, more hours of patching will be associated with higher levels of reported caregiver stress measured at a later time point (Fig. 1a), and (2) higher levels of reported caregiver stress will be associated with fewer hours of patching at a later time point (Fig. 1b). These questions were included as secondary outcomes in the original design of the IATS and not posthoc analyses.

## Methods

### Subjects and Methods

The overall design of the IATS and results of the visual acuity assessment at 4.5 years of age have been published.^11^,^12^ Briefly, the IATS was a multicenter randomized controlled trial comparing two treatments for visually significant UCC in children aged 6 months or younger: removal of the cataractous lens followed by contact lens (CL) correction of aphakia or IOL implantation at the time of lens extraction. Children were excluded if the eye had a corneal diameter less than 9 mm; the intraocular pressure was 25 mm Hg or greater; there was persistent fetal vasculature (PFV) causing stretching of the ciliary processes or a tractional retinal detachment; retinal or optic nerve disease, or signs suggestive of uveitis; the child was preterm; the fellow eye had ocular disease that might reduce its visual potential; the child had a medical condition known to limit the ability to obtain visual acuity at 12 months or 4 years of age; or follow-up of the child was not feasible. Written informed consent from caregivers was obtained prior to participation. The study was approved by the institutional review boards of all participating institutions and was in accordance with the tenets of the Declaration of Helsinki.

### Prescribed Patching and Visual Correction

Patching was prescribed for all children until age five. Starting the second week after cataract surgery, caregivers were instructed to have the child wear an adhesive occlusive patch over the fellow eye 1 hour daily per month of age until the child was 8 months old. Thereafter, caregivers were told to patch their child 50% of waking hours. Patches were provided to patients at no cost.

Refractive correction was prescribed for all aphakic children 100% of waking hours. Within a week after cataract surgery, aphakic patients were fitted with a silicone elastomer (Silsoft; Bausch & Lomb, Rochester, NY) or a rigid gas permeable CL with a 2.0-D overcorrection to provide a near-point correction. A spare lens was provided to ensure that optical correction was available in the event of loss or damage. Both daily wear and extended wear protocols were acceptable. At age 2 years, the eye was corrected to emmetropia using a CL, and spectacles were prescribed with +3.0 D bifocal lens for near focus.

For pseudophakic infants, spectacles were prescribed by the 1-month postoperative visit if any of the following conditions existed: hyperopia >1.0 D, myopia > 3.0 D, or astigmatism > 1.5 D. In children younger than 2 years, the aim was to correct the refractive error to 2.0 D of myopia; thereafter the aim was emmetropia at distance with a near correction of +3.0 D. The phakic eye for both groups was corrected with spectacles under any of the following conditions: hyperopia >5.0 D, myopia >5.0 D, astigmatism >1.5 D, or refractive esotropia. The aim was to correct the refractive error to the range of 0 to +3.0.

### Stress Measures

The Parenting Stress Index (PSI)^15^ was used to assess stress associated with the parenting role. The PSI was completed as part of a self-administered questionnaire completed by the primary caregiver in his/her preferred language (English, Spanish, or Portuguese) three times during one of the quarterly clinic visits: (1) 3 months after surgery (3 months), (2) 3 months after the child's vision was assessed at 12 months of age (approximately 15 months of age), and (3) at 51 months of age (4.25 years). The caregiver placed the questionnaire in a sealed envelope and returned it to the study staff at the clinical center who mailed it to the Data Coordinating Center (DCC) at Emory University.

The PSI is a well-validated, age-normed 120-item self-report measure of parenting stress. Respondents rate their agreement with each statement on a five-point scale (e.g., “My child is much more active than I expected,” “I feel trapped by my responsibilities as a parent”). The scale yields two factor-based scores, a Child Domain score (with subscales Distractibility/Hyperactivity, Adaptability, Reinforces Parent, Demandingness, Mood, Acceptability) and a Parent Domain score (with subscales Competence, Isolation, Attachment, Health, Role Restriction, Depression, Spouse), as well as a Total Stress score. A Life Stress score, derived from 19 items not included in the Total Stress score assesses respondents' exposure to stressful life events (e.g., death of a relative, loss of a job) outside the parent–child relationship. The PSI is interpreted via age-based percentile scores derived from the frequency distribution of the normative sample (1-month to 12-year olds), with higher scores indicating higher levels of stress. Many studies confirm the reliability and validity of the Total Stress score.^15^

### Assessment of Adherence to Patching

Adherence to prescribed patching was reported by caregivers in semi-structured telephone interviews. The interviews were completed quarterly, starting 3 months after surgery. An additional interview was conducted prior to the 12-month visual acuity assessment to obtain adherence information in close proximity to the time when primary outcome was assessed. At each interview, the caregiver reported specific times when the patch was applied and either fell off or was removed over the previous 48 hours. Caregivers also reported use of glasses and/or CL, and when their child was asleep. The timing of the interviews was determined using an algorithm that distributed the preferred day of the call evenly throughout the week, including weekends. Caregivers were not informed in advance about when they would be contacted to complete an interview. The interviews were conducted in the caregiver's preferred language (English, Spanish, or Portuguese) by one of three trained interviewers so that the caregiver spoke with the same person on each occasion. The English-speaking interviewer performed the vast majority of interviews (>95%). Interviewers were located centrally, at the DCC, and masked to treatment assignment to minimize the possibility that the respondent would exaggerate adherence or that the interviewer's interpretation of the information would be biased by knowledge of the child's visual acuity. However, it was not possible to ensure that the interviewer remained masked to treatment group over time. Additionally, the same information was obtained annually through a 7-day prospective patching diary completed 2 months after surgery and 1 month following each of the child's birthdays. The Cronbach's alphas suggest that the reliability of the diary and the telephone interview for collecting data on patching adherence collected were acceptable in the first year after surgery (α = 0.69) and good thereafter (α = 0.85–0.87 2, 3, and 4 years after surgery).^16^

Based on information reported in the interview, we calculated the average number of waking hours each day that the child was occluded over six specific time periods: (first) prior to the first caregiver questionnaire at 3 months postsurgery; (second) the 6 months after the first caregiver questionnaire; (third) the 6 months immediately prior to the second caregiver questionnaire administered around 15 months of age; (fourth) the 6 months immediately after the second caregiver questionnaire; (fifth) the 6 months before the third caregiver questionnaire was administered at 4.25 years of age; and (sixth) the 6 months after the third administration of the caregiver questionnaire. For a substantial number of children (*n* = 71) assessments included in the second window (i.e., the 6 months after the first caregiver questionnaire) were also included the third window (i.e., the 6 months before the second caregiver questionnaire) because these two questionnaires were typically administered 6 to 9 months apart. However, this is of limited concern for the current analyses since these two variables were never included in the same analytic model. All of the other adherence assessments were included only in one of the six periods.

### Analytic Methods

Statistical analyses were conducted using SPSS 21 (IBM Corp., 2012, IBM SPSS Statistics for Windows, Version 21.0., Armonk, NY) and SAS 9.2 (SAS Institute, Inc., Cary, NC). Pearson's correlation coefficients and linear regression were used to estimate the association between patching and caregiver stress. Statistical significance was defined at α = 0.05. A priori we defined the following as potential confounders because of their likely association with both caregiver stress and patching: treatment (IOL versus CL), child sex, age at surgery (<49 days versus 49–208 days), socioeconomic status (private insurance versus other payment), and adverse events (any/none) occurring up to 12 months prior to each stress assessment; these variables were included as covariates in regression models. Additionally, since adherence at one time point is likely associated with prior adherence, for models assessing whether higher stress is associated with fewer hours of patching in the subsequent 6 months, we also controlled for the average number of hours of patching in the preceding 6 months.

## Results

The IATS enrolled 114 children; 57 randomized to each treatment group (Fig. 2). At age 4.5 optotype visual acuity was assessed in 112 children. For the current analyses, we excluded three participants with adverse outcomes limiting their visual potential (one with Stickler's syndrome, one with phthisis, and one with endophthalmitis) and who were told not to patch subsequent to these events. The demographic characteristics of the remaining participants for whom parenting stress data were available are shown in Table 1.

**Table 1:** Characteristics of Participants in the IATS

	%	Number (*n* = 111)
IOL	50.5	56
Surgery performed at 49–210 days of age^a^	54.1	60
Female	54.1	60
Private insurance	62.2	69
White race	84.7	94
≥1 Adverse event before first stress assessment	36.9	41
≥1 Adverse event before second stress assessment	53.2	59
≥1 Adverse event in the 12 months prior to the third stress assessment	13.5	15

a Versus 28–48 days of age.

The average hours of occlusion at each time point was based on one to five telephone interviews depending on the age at surgery and the time point (Table 2). On average, caregivers reported patching their child between 3 and 4.5 hours per day. However, at all time periods, there was substantial variation in the amount of time that parents reported patching their child. The caregiver questionnaires were completed by 106 caregivers 3 months after surgery, by 97 caregivers after the 12-month visual acuity assessment, and by 96 caregivers at 4.25 years of age. Consistent with our previous reports, overall levels of parenting stress were similar in the first, second, and third caregiver questionnaires (*P* = 0.438) (Table 3).

**Table 2:** Number of Completed Assessments of Adherence to Patching and Reported Levels of Patching in the IATS by Time Period

Time Period	Number of Assessments of Patching *n*	Daily Hours of Patching	
		Mean Hours ± SD	IQR (25th%, 75th%)
Before first stress assessment			
* 0*^a^	*21*		
1	66	3.8 ± 1.5	(2.8, 4.7)
2	24		
6 months after first stress assessment			
* 0*	*6*		
1	10	4.3 ± 2.0	(3.0, 5.6)
2	57		
3+	38		
6 months before the second stress assessment			
* 0*	*15*		
1	1	4.1 ± 2.1	(2.4, 5.6)
2	22		
3+	73		
6 months after the second stress assessment			
* 0*	*16*		
1	5	3.5 ± 2.2	(1.9, 5.4)
2	62		
3+	28		
6 months before the third stress assessment			
* 0*	*26*		
1	13	3.4 ± 2.7	(1.1, 4.9)
2	20		
3+	42		
6 months after the third stress assessment			
* 0*	*30*		
1	17	3.2 ± 2.9	(0.8, 5.0)
2	63		
3	1		

a Individuals with 0 assessments, indicated by italics, in a time period are not included in analyses using that variable.

**Table 3:** Reported Parenting Stress Levels by Timing of Assessment

Assessment	*n*	PSI – Total		PSI – Parent Domain	
		Mean ± SD	IQR	Mean ± SD	IQR
First^a^	106	204.4 ± 36.0	(179.8–228.3)	111.9 ± 23.5	(94.0–128.0)
Second^b^	97	200.2 ± 33.9	(173.0–227.0)	107.4 ± 22.2	(89.0–124.5)
Third^c^	96	203.6 ± 41.4	(179.3–232.8)	108.6 ± 24.5	(93.0–123.8)

a First assessment completed at the 3-month postsurgical visit.

b Second assessment completed at the first three quarterly clinic visits after the visual acuity assessment at 12 months of age.

c Third assessment completed at the visit at 4.25 years of age.

**Table 3:** Extended

Assessment	PSI – Child Domain		Life Stress	
	Mean ± SD	IQR	Mean ± SD	IQR
First^a^	92.5 ± 16.8	(80.5–103.0)	8.6 ± 7.4	(3.0–12.0)
Second^b^	92.9 ± 14.9	(82.0–102.0)	7.7 ± 7.6	(2.0–10.0)
Third^c^	94.7 ± 19.6	(79.5–107.5)	8.7 ± 7.9	(4.0–12.0)

### Are Higher Levels of Patching Associated With Higher Levels of Caregiver Stress Measured at a Later Time Point?

Parents reporting more hours of patching in the preceding 6 months tended to report somewhat lower levels of parenting stress rather than higher levels as we had hypothesized (Table 4). These observed differences accounted for little variation in the reported levels of parenting stress and were statistically significant only for the total and parent domain of the PSI assessed 3 months after surgery.

**Table 4:** Average Change (95% CI) in Reported Parenting Stress Levels Associated With a 1-Hour Increase in Reported Average Hours of Patching in the Prior Period^a^

	Parenting Stress Reported 3 Months After Surgery (*n* = 90)		Parenting Stress Reported at 15 Months of Age (*n* = 96)	
	Crude	Adjusted^b^	Crude	Adjusted^c^
PSI – Total	−5.3 (−10.1 to −0.4)*	−4.9 (−22.2 to 9.7)*	−2.7 (−6.0 to 0.5)	−2.7 (−6.1 to 0.7)
PSI – Parent domain	−3.8 (−6.9 to −0.7)*	−3.5 (−6.7 to −0.3)*	−1.1 (−3.3 to 1.0)	−1.3 (−3.6 to 0.9)
PSI – Child domain	−1.4 (−3.8 to 0.9)	−1.4 (−3.8 to 1.0)	−1.6 (−3.0 to −0.2)*	−1.4 (−2.8 to 0.1)
Life stress	−0.6 (−1.6 to 0.3)	−0.7 (−1.7 to 0.3)	−0.4 (−1.2 to 0.3)	−0.3 (−1.0 to 0.5)

a Prior 3 months for the first assessment of parenting stress; prior 6 months for the subsequent two assessments of parenting stress.

b Adjusted for treatment, age at surgery, private insurance, and adverse events.

c Adjusted for treatment, age at surgery, private insurance, and adverse events.

d Adjusted for treatment, age at surgery, private insurance, and adverse events.

* *P* < 0.05.

**Table 4:** Extended

	Parenting Stress Reported at 4.25 Years of Age (*n* = 95)	
	Crude	Adjusted^d^
PSI – Total	−1.2 (−4.7 to 2.2)	−1.8 (−5.3 to 1.8)
PSI – Parent domain	−1.0 (−3.0 to 1.0)	−1.2 (−3.3 to 0.9)
PSI – Child domain	−0.3 (−1.9 to 1.3)	−0.6 (−2.2 to 1.01)
Life stress	−0.7 (−1.4 to −0.0)	−0.7 (−1.3 to −0.0)

### Are Higher Levels of Caregiver Stress Associated With Fewer Hours of Patching at a Later Time Point?

Our second hypothesis was that caregivers reporting high levels of parenting stress would be less adherent to subsequent prescribed patching. As hypothesized, we observed that caregivers reporting higher levels of parenting stress 3 months after surgery reported fewer hours of patching in the subsequent 6 months. This association remained statistically significant for the PSI, as a whole, for both the parent and child subdomains of the PSI and for the life stress scale, even after controlling for treatment, age at surgery, private insurance, prior adverse events, and previous hours of patching. In order to translate the observed regression coefficients into time, we estimated the change in predicted hours per day of patching resulting from going from the 25th percentile of reported stress to the 75th percentile of reported stress (Table 5). This analysis shows, for example, that caregivers reporting parenting stress levels at the 75th percentile of the distribution reported patching approximately 52 minutes per day (0.87 hours) less patching than caregivers reporting stress levels at the 25th percentile. Higher levels of stress reported in the second caregiver questionnaire were also associated with fewer hours of patching in the subsequent 6 months. However, after controlling for previous patching and other covariates, the association was no longer statistically significant. There was little evidence that higher stress levels reported on the third caregiver questionnaire were associated with subsequent reported adherence to occlusion.

**Table 5:** Changes in Reported Average Hours of Patching Per Day in the Subsequent 6 Months Associated With a Change in Reported Stress Level From the 25th Percentile to the 75th Percentile

	First Stress Assessment^a^ (*n* = 90)		Second Stress Assessment^b^ (*n* = 94)	
	Crude	Adjusted^d^	Crude	Adjusted^e^
PSI – Total	−1.16 (−1.70 to −0.63)	−0.87 (−1.35 to −0.34)	−0.81 (−1.51 to −0.11)	−0.38 (−0.92 to 0.16)
PSI – Parent domain	−1.22 (−1.84 to −0.65)	−0.88 (−1.43 to −0.34)	−0.75 (−1.46 to −0.04)	−0.43 (−0.96 to 0.14)
PSI – Child domain	−.83 (−1.37 to −0.29)	−0.61 (−1.10 to −0.09)	−0.68 (−1.29 to −0.06)	−0.25 (−0.74 to 0.25)
Life stress	−0.63 (−1.17 to −0.08)	−0.41 (−0.91 to −0.07)	−0.14 (−0.65 to 0.36)	−0.19 (−0.20 to 0.59)

a Completed approximately 3 months postsurgery.

b Completed at the first quarterly clinic visit after the 12-month visual acuity assessment. Usually completed between 12 and 15 months of age.

c Completed around 4.25 years of age.

d Adjusted for treatment, age at surgery, private insurance, adverse events prior to the first stress assessment, and patching prior to the first stress assessment.

e Adjusted for treatment, age at surgery, private insurance, adverse events prior to the visual acuity assessment, and patching in the 6 months prior to the second stress assessment.

f Adjusted for treatment, age at surgery, private insurance, adverse events in the 12 months prior to the third stress assessment, and patching in the 6 months prior to the third stress assessment.

* *P* < 0.05.

**Table 5:** Extended

	Third Stress Assessment^c^ (*n* = 77)	
	Crude	Adjusted^f^
PSI – Total	−0.05 (−0.86 to 0.75)	0.27 (−0.21 to 0.75)
PSI – Parent domain	−0.18 (−0.98 to 0.62)	0.25 (−0.22 to 0.71)
PSI – Child domain	0.17 (−0.76 to 1.06)	0.28 (−0.25 to 0.84)
Life stress	−0.69 (−1.29 to −0.09)	−0.11 (−0.50 to 0.27)

## Discussion

There was no evidence that adherence to prescribed patching substantially increases parenting stress in caregivers of infants and young children who have been treated for UCC. In contrast, there was some evidence that higher levels of parenting stress were associated with reductions in patching, particularly in the first months after surgery, even after accounting for prior adherence to patching. Three months after surgery, caregivers reporting the lowest stress levels reported approximately 45 minutes more of patching per day than did those reporting the highest stress levels (Table 5). The association was only statistically significant at the first time point, and was not observed during the fifth year of life. Thus, we believe that after patching habits have been established, parenting stress has minimal impact on the amount of time that a child is occluded. Perhaps these associations are stronger at the first stress assessment than at subsequent stress assessments because this is the time period when patching habits become formed and are associated with initial improvements in visual acuity.^17^,^18^ However, as time since surgery, prescribed patching, and age are strongly co-linear in our data, we cannot fully distinguish the impact of time since patching started and age of the child.

Although the observed associations are relatively weak and are limited to the first assessment of stress, which was completed shortly after surgery, these results are consistent with associations found between high levels of parenting stress and poor illness management among 8- to 13-year-old children with poor asthma control.^19^ They are also aligned with a prospective study of medication adherence in a longitudinal study of 2- to 12-year-old children with newly diagnosed epilepsy.^20^ However, our findings are not consistent with results from other studies. For example, parenting stress assessed with the short form of the PSI was not related to objective measures of medication adherence in cross-sectional studies of 2- to 5-year olds with asthma^21^ or 1- to 13-year-old children perinatally infected with HIV,^22^ or to treatment adherence for 2- to 8-year olds with type 1 diabetes.^23^ Therefore, the effects that we observe here could reflect the fact that parenting stress plays a different role in caregivers' adherence to prescribed medical treatment in these very young children undergoing cataract surgery than it may for children at later ages or those with different medical conditions.

This is one of the first studies to have examined the potential role of parenting stress on adherence to patching in children with UCC. Further, we were able to examine this question at three different time points and we were able to control for adherence to patching in earlier time points. However, we have no data on parenting stress for the period from approximately 15 months of age until after age 4; a period during which there are significant developmental changes, and during which there may be substantial changes in the caregiver–child relationship. Additionally, we have missing data on a relatively large proportion of participants, particularly at the final time point. Further, although multiple adherence assessments were included in all time windows for most families, there are day-to-day variations in hours of patching that were achieved; therefore, the average amount of patching reported may be affected by random error.

Finally, daily hours of patching in one time period is strongly correlated with later patching. For example, the correlation coefficient between reported hours of patching prior to the first adherence assessment and reported hours of patching after the first assessment was 0.46. The association was even stronger for adherence surrounding the second (0.68) and third (0.84) stress assessments. Thus, we may have limited our ability to detect the impact of stress on subsequent patching, and thus, it is possible that the impact of stress on patching is greater than we report here, particularly in the fifth year of life.

We chose to evaluate the average hours per day of patching reported by caregivers rather than using the percent of prescribed patching achieved for clarity of presentation and interpretation. However, we recognize that these two may not be equivalent because until age 8 months, parents were told to patch their child 1 hour per day per month of life; thereafter they were told to patch the child 50% of waking hours. Further the number of waking hours during which the child is patched generally decreases with age, as does the number of hours of sleep reported. For example, after the first stress assessment, on average children were reported to sleep about 12.6 hours per day (interquartile range [IQR] = 11.7–13.5); while after the third assessment, children were reported to sleep an average of less than 11 hours per day (10.8, IQR = 10.2–11.3). Even so, the results of the analyses using percent of prescribed patching and average hours per day were similar, and the two variables were strongly correlated with each other at all time points (Pearson's correlation coefficient = 0.76 for the period prior to the first stress assessment and >0.95 at all other time points). Therefore, we have chosen to describe the association between reported stress and hours per day of patching for ease of interpretation.

In sum, parenting stress may contribute to poor adherence to prescribed patching in children treated for UCC, particularly in the early months following surgery. Clinicians should be aware of factors that may impede a family's ability to adhere to prescribed patching guidelines. Although our data suggest that the association between parenting stress and patching adherence may not persist, the first year following surgery is a critical time period for caregivers' efforts to establish patching habits. Routines, for example around patching, are believed to “serve as setting events for child compliance and aid in the development of rule-governed behavior, which in turn can function to create a behavioral history of rule following and thereby impact general child behavior management” and routines may reduce caregiver stress by providing structure.^24^ Also, to the extent that patching in the first months following cataract extraction facilitates visual development, better visual acuity in the first months following cataract surgery may help reduce resistance to patching later when children may be more able to physically and verbally resist caregivers' efforts to patch. Thus, it may be prudent for clinicians to assess parenting stress in the caregivers of children treated for UCC and provide additional support for patching efforts to those caregivers who report high levels of stress. Given the importance of adherence to prescribed patching following surgery for UCC, research into the factors that facilitate caregivers' patching efforts should continue.

## Supplementary Material

Supplement 1
