# Supplementary material for: Parenting Stress and Adherence to Occlusion Therapy in the Infant Aphakia Treatment Study: A Secondary Analysis of a Randomized Clinical Trial
Source: Transl Vis Sci Technol. 2019 Jan 2;8(1):3. doi: 10.1167/tvst.8.1.3 (PMC6322710; doi:10.1167/tvst.8.1.3)
Supplement: Supplement 1 [file tvst-07-06-33_s01.doc]

**Appendix : The Infant Aphakia Treatment Study Group**

**Administrative Units**

**Clinical Coordinating Center (Emory University):** Scott R. Lambert, MD (Study Chair); Lindreth DuBois, MEd, MMSc (National Coordinator)

**Contact Lens Committee:** Buddy Russell, COMT; Michael Ward, MMSc

**Data and Safety Monitoring Committee**: Robert Hardy, PHD (Chair); Eileen Birch, PhD; Ken Cheng, MD; Richard Hertle, MD; Craig Kollman, PhD; Marshalyn Yeargin-Allsopp, MD (resigned); Cyd McDowell; Donald F. Everett, MA (ex officio)

**Data Coordinating Center (Emory University):** Michael Lynn MS (Director), Betsy Bridgman, BS; Marianne Celano PhD; Julia Cleveland, MSPH; George Cotsonis, MS; Carolyn Drews-Botsch, PhD; Nana Freret, MSN; Lu Lu, MS; Azhar Nizam, MS; Seegar Swanson; Thandeka Tutu-Gxashe, MPH

**Eye Movement Reading Center (University of Alabama, Birmingham and Retina Foundation of the Southwest, Dallas, TX):** Claudio Busettini, PhD, Samuel Hayley, Joost Felius, PhD

**Medical Safety Monitor:** Allen Beck, MD

**Program Office (National Eye Institute)**: Donald F. Everett, MA

**Steering Committee:** Scott R. Lambert, MD; Edward G. Buckley, MD; David A. Plager, MD; M. Edward Wilson, MD; Michael Lynn, MS; Lindreth DuBois, Med MMSc; Carolyn Drews-Botsch, PhD; E. Eugenie Hartmann, PhD; Donald F. Everett, MA

**Vision and Developmental Testing Center (University of Alabama, Birmingham)**: E. Eugenie Hartmann, PhD (Director); Anna K Carrigan, MPH; Clara Edwards;

**Participating Clinical Centers (In order by the number of patients enrolled):**

**Medical University of South Carolina; Charleston, South Carolina (14):** M. Edward Wilson, MD; Margaret Bozic, CCRC, COA

**Harvard University; Boston, Massachusetts (14):** Deborah K. Vanderveen, MD; Theresa A. Mansfield, RN; Kathryn Bisceglia Miller, OD

**University of Minnesota; Minneapolis, Minnesota (13):** Stephen P. Christiansen, MD; Erick D. Bothun, MD; Ann Holleschau, B.A.; Jason Jedlicka, OD; Patricia Winters, OD; Jacob Lang, O.D.

**Cleveland Clinic; Cleveland, Ohio (10):** Elias I. Traboulsi, MD; Susan Crowe, BS, COT; Heather Hasley Cimino, OD

**Baylor College of Medicine; Houston, Texas (10):** Kimberly G. Yen, MD; Maria Castanes, MPH; Alma Sanchez, COA; Shirley York

**Emory University; Atlanta, Georgia (9):** Scott R. Lambert, MD; Amy K. Hutchinson, MD; Lindreth Dubois, Med, MMSc; Rachel Robb, MMSc; Marla J. Shainberg, CO

**Oregon Health and Science University; Portland, Oregon (9):** David T Wheeler, MD; Ann U. Stout, MD; Paula Rauch, OT, CRC; Kimberly Beaudet, CO, COMT; Pam Berg, CO, COMT

**Duke University; Durham, North Carolina (8):** Edward G. Buckley, MD; Sharon F. Freedman, MD; Lois Duncan, BS; B.W. Phillips, FCLSA; John T. Petrowski, OD

**Vanderbilt University: Nashville, Tennessee (8)**: David Morrison, MD; Sandy Owings COA, CCRP; Ron Biernacki CO, COMT; Christine Franklin, COT

**Indiana University, Indianapolis, Indiana (7):** David A. Plager, MD; Daniel E. Neely, MD; Michele Whitaker, COT; Donna Bates, COA; Dana Donaldson, OD

**Miami Children’s Hospital, Miami, Florida (6):** Stacey Kruger, MD; Charlotte Tibi, CO; Susan Vega

**University of Texas Southwestern; Dallas, Texas (6):** David R. Weakley, MD; David R. Stager Jr M.D.; Joost Felius, PhD; Clare Dias, CO; Debra L. Sager; Todd Brantley, OD

**Case Western Reserve, Cleveland, Ohio (1)**: Faruk Orge, M.D.
